# Dr. George William Reynolds

**Published:** 1911-11

**Authors:** 


### OBITUARY

---

Dr. George William Reynolds, a graduate of the University of Buffalo, 1880, Chief of the gynecologic staff, St. Joseph Hospital, Chicago, died in that institution, September 9, 1911, aged 57, of chronic interstitial nephritis.

